# Preventing Financial Elder Abuse: A Critical Commentary on Harmonisation of Enduring Power of Attorney Laws

**DOI:** 10.1111/ajag.70122

**Published:** 2026-02-04

**Authors:** Nola Ries, Eugenia Tsihlis, Teresa Somes

**Affiliations:** ^1^ Faculty of Law University of Technology Sydney New South Wales Australia

## Abstract

Ending the abuse of older people in Australia is a national policy priority. Harmonisation of financial enduring power of attorney (FEPOA) legislation is regularly called for to protect against financial elder abuse. A FEPOA is a legal instrument by which an adult appoints one or more people to manage their legal obligations, money and property, particularly during periods of incapacity. Although older Australians are urged to make FEPOAs, and a majority of those over age 65 report having one, these instruments are implicated in an estimated 50%–85% of cases of financial elder abuse. This commentary offers a critical perspective on legislative harmonisation as a policy intervention to prevent financial abuse committed via FEPOA arrangements. We address three main issues. First, we explain several approaches to harmonisation, illustrated with examples from different areas of law‐making, noting that harmonisation is often a slow process that does not achieve uniformity. Second, we argue that deficiencies in knowledge and practices that create risks of financial abuse via FEPOAs will not be cured by harmonisation alone. Third, we highlight that the legislative variation across states and territories provides opportunities for innovation in research. Further investment in high‐quality research is essential to guide effective legislative and compliance strategies to prevent the financial abuse of older people.

## Policy Impact

Calls for harmonising financial enduring power of attorney (FEPOA) laws aim to protect older adults from abuse. However, we lack evidence on which legal rules deter FEPOA abuse and warrant inclusion in nationally consistent laws. Knowledge gaps and risky practices must be addressed separately from the slow law reform process.

## 1 Introduction

Ending the abuse of older people in Australia is a national policy priority [1]. Harmonisation of financial enduring power of attorney (FEPOA) legislation is regularly called for to protect against financial elder abuse. A FEPOA is a legal instrument by which an adult appoints one or more people to manage their legal obligations, money and property, particularly during periods of incapacity. Although this fundamental purpose of a FEPOA is consistent across the country, state and territory laws vary, including differences in requirements for witnessing, execution and activation of FEPOAs, specification of attorneys' duties, and oversight and accountability mechanisms [2]. These variations reflect different policy approaches to balancing autonomy, protection and administrative efficiency, but can be confusing to navigate, particularly when dealing with interstate assets and arrangements.

Older Australians are urged to make a FEPOA and a majority of those over age 65 report having one [3, 4]. In theory, this legal instrument offers peace of mind by giving older people choice and control over how their assets are managed in the future. In practice, FEPOAs are implicated in an estimated 50%–85% of cases of financial elder abuse [5, 6] and strategies to prevent this form of abuse are urgently needed.

Harmonisation of FEPOA laws is regularly called for as a necessary reform. In this article, we offer a critical perspective on legislative harmonisation as a policy intervention to prevent the abuse of older people. We address three main issues. First, to foster informed discussion of harmonisation, we explain several approaches to harmonisation, illustrated with examples from different areas of law‐making. The history of harmonisation is that it is a slow process and often does not achieve uniformity across the country. Second, we consider how the letter of the law is distinct from the behaviours it seeks to promote within social systems [7] and argue that deficiencies in knowledge and practices that create risks of financial abuse via FEPOAs will not be cured by harmonisation alone. Third, we draw attention to the under‐acknowledged benefits of legislative variation across states and territories—namely, that it provides opportunities for innovation in research. Legislation is a key intervention to achieve policy goals, yet the impacts of law are remarkably under‐researched [8]. Further investment in high‐quality research is essential to determine what features of laws should be adopted in a harmonised approach, and to guide other strategies that support compliance with the law.

## 2 Calls for Harmonisation

In 2017, the Australian Law Reform Commission's major report, *Elder Abuse—A National Legal Response*, called for ‘nationally consistent laws’ governing enduring representatives, along with the development of a ‘short, simple’ model enduring document to cover appointments for financial and other matters, including medical, personal and lifestyle decisions [9]. This legislative harmonisation was recommended as a precondition to other reforms, such as a national register of enduring appointments.

Harmonisation of FEPOAs was prioritised in the *National Plan to Respond to the Abuse of Older Australians 2019–2023*, given concerns about the abuse of these instruments to financially exploit older people [10]. In 2023, the Commonwealth Attorney‐General proposed key elements of a model FEPOA document and ‘potential improvements to arrangements for recognising EPOAs interstate’ [2]. The *Evaluation of the National Plan to Respond to the Abuse of Older Australians*, released in July 2024, described as an unresolved problem the ‘State by state inconsistencies in laws and processes pertaining to Powers of Attorney’ [11, p. 125]. Harmonisation of laws was again cited as a solution and the report noted frustrations over the slow progress towards harmonising laws pertaining to enduring representatives [11, p. 127].

In September 2024, the Australian Human Rights Commission (AHRC) released its *Empowering Futures* report with findings from a national survey on public knowledge and use of FEPOAs [12]. The report renewed the call for ‘national consistency in FEPOA laws as a priority’, with law reform again presented as a precursor to a national education campaign and a register of enduring documents [12, p. 7]. The proposed *National Plan to End the Abuse and Mistreatment of Older People 2024–2034*, released by the Commonwealth Attorney‐General's Department, asserts that ‘[a]chieving greater consistency in EPOA laws continues to be a priority’ [13, p. 49].

These developments all point to an aspirational intention to bring greater ‘consistency’ or harmonisation to the laws that govern FEPOA across Australia. However, history has shown that legislative harmonisation is likely to be a protracted exercise.

## 3 What Does Harmonisation Mean?

Harmonisation of laws can be conceptualised on a continuum—from compatibility, to consistency, to uniformity [14]. Compatibility can be achieved where jurisdictions enact agreed minimum standards in their legislation and/or by mutually recognising legal documents or determinations [14]. Some degree of compatibility already exists across FEPOA laws as they share fundamental elements. For example, the person making the appointment (the ‘principal’) must have the requisite capacity to do so. They can appoint one or more attorneys and impose conditions or limitations on the scope of the attorney's authority. Appointed attorneys must act in the interests of the principal and FEPOAs can be changed or revoked. Additionally, some jurisdictions already provide for mutual recognition, meaning that a FEPOA made in one state or territory is recognised as legally valid in other jurisdictions [2]. Consistency can be achieved by jurisdictions adopting a common set of legislative rules and administrative processes. Uniformity would apply the same law in all jurisdictions or refer powers to the Commonwealth to legislate in the area [14].

Table 1 explains four approaches to legislative harmonisation and their benefits and drawbacks, with illustrative examples from different areas of law. In many instances, harmonisation efforts do not achieve uniform laws across Australia, as jurisdictions may wish to retain features of their own laws that provide for higher standards or locally preferred processes. Moreover, harmonisation initiatives are often incremental and protracted. One example of particular relevance to older Australians concerns succession laws, which govern the distribution of assets following a person's death. In the early 1990s, Attorneys‐General across Australia agreed to work towards uniform succession laws, covering legal rules in regard to wills, intestacy, family challenges to wills and deceased estate administration. This was an ambitious task, and recent analysis concludes that ‘the level of uniformity across the jurisdictions remains low’ [18].

**TABLE 1:** Types of legislative harmonisation.

Definitions provided by the PCC^a^	Example where this harmonisation taken place [15]	Benefits of the harmonisation activity [16, 17]	Drawbacks of the harmonisation activity
(a) National applied laws legislation (or ‘template’ legislation), that is, legislation enacted in one jurisdiction and applied (as in force from time to time) by other participating jurisdictions as a law of those other jurisdictions	Electronic conveyancing, consumer protection, agricultural and veterinary chemicals	If the Commonwealth leads the way through providing the template legislation, it is more likely to produce a streamlined result and sustainable uniformity	Can be extremely complicated as jurisdictions can ‘apply’ the law in different ways, as it may be on a ‘adopt as amended from time to time’ or ‘as is’, that is automatically updated in another state if the ‘template’ is amended, or requiring changes to be passed separately through Parliamentary processes at state level
(b) National model legislation, that is, legislation that is drafted as model legislation and that is enacted in participating jurisdictions (with any local variations that are necessary to achieve the agreed uniform national policy when the legislation forms part of the local law)	Work health and safety (WHS) Uniform Evidence Act	Harmonisation in this way can be made simpler through oversight of a single government statutory body (in the case of WHS this is Safe Work Australia). Safe Work Australia develops national policy relating to WHS and the states and territories are responsible for enforcing the laws	There can be differences in how the law is implemented and amendments are not automatically applied to a jurisdiction's own version Risk of ‘hold‐out’ states, for example, Queensland and South Australia have refused to accept the model evidence law, instead maintaining their existing Acts and may adopt individual elements of the uniform law. It was only in August 2024 that Western Australia introduced a new Act based on the uniform law to Parliament
(c) Legislation of the States referring legislative power to the Commonwealth	Business names registration, corporations legislation, counterterrorism legislation	Efficient to administer, easier to understand across interstate borders, no need for a patchwork of merely ‘complementary’ laws across jurisdictions	Although the closest to complete uniformity between laws, it is not guaranteed. For example, Corporations legislation still has minor variations in some states
(d) Legislation of a particular jurisdiction that is identified as legislation that will be followed in other jurisdictions (in this case, the matter can be added to the PCC agenda for settling as model legislation or for informal comments from other PCC members to promote uniform legislation in appropriate cases)	Health practitioner regulation	Can be an influential starting point for harmonisation that then falls into one of the categories named above as it will be driven by the PCC directly	If it becomes an ‘applied’ law framework, complexities can arise as stated above. For example, New South Wales diverged from the model law by keeping its own provisions on fitness‐to‐practice concerns

^a^ The legislative drafting offices in Australia and New Zealand are represented on the Parliamentary Counsel's Committee, which is a forum for developing national uniform laws [15].

## 4 Harmonisation of FEPOA Laws

Calls for harmonisation of FEPOA laws favour a model legislation approach, whereby jurisdictions would adopt a common set of core legislative provisions. Model provisions have been proposed by the Commonwealth Attorney‐General's Department [2], with responses or suggestions from other bodies, such as the Law Council of Australia [19] and the Queensland Public Advocate [20]. The model provisions would enhance consistency in regard to procedures for making FEPOAs, eligibility to be appointed to the attorney role, duties of attorneys, cessation or revocation of an appointment, offences and remedies. Under this approach to harmonisation, jurisdictions could retain features of their existing laws. Indeed, the Attorney‐General's consultation paper acknowledges that ‘retaining jurisdiction‐specific approaches in certain areas of financial EPOA law is necessary and appropriate’ [2, p. 5]. Reasons for doing so are that some jurisdictions may have existing legislative provisions that are viewed as offering greater safeguards than the proposed model rules. Some jurisdictions integrate several kinds of enduring representative appointments in one law—financial, medical, personal, lifestyle—with appointment forms that reflect this approach rather than being specific to FEPOA.

Uniformity of FEPOA laws across Australia and a standard national form are highly unlikely any time soon; at best, incremental steps may occur towards improving consistency [1]. Even if model legislative provisions and a FEPOA template were adopted across the country, such harmonisation would offer a degree of ‘textual uniformity [and] not necessarily deliver similar outcomes or experiences’ [14, p. 334].

We do not oppose harmonisation efforts, but our position is such work should be evidence‐informed and not raise unrealistic expectations that legislative consistency alone will prevent financial elder abuse. Nor should the pursuit of harmonisation take priority over interventions to ameliorate the fundamental knowledge and practice deficits that exist independent of legislative variation. Pursuing harmonisation without understanding which legal provisions are most effective may result in adopting model laws that fail to incorporate the most protective features, or worse, that inadvertently create new risks. We now turn to a key outcome sought to be achieved through harmonisation—the prevention of financial abuse of older people.

## 5 Preventing Abuse via FEPOA

Legislative harmonisation cannot be understood ‘in isolation from the policy aims it is designed to achieve’ [14, p. 326]. A fundamental aim of harmonising FEPOA laws is to prevent financial abuse by people who gain access to and control over an older person's money and other assets via a FEPOA appointment. Other aims relate to improving remedial actions, including access to compensation, in the event an attorney has misused or abused their powers. Here, we focus on primary prevention [21]. Greater consistency in the text of the laws is not a standalone solution to preventing financial elder abuse. Known deficiencies in knowledge and practices that create risk factors for FEPOA abuse must be addressed independently of harmonisation.

### 5.1 Knowledge Deficits

Existing research points to fundamental public knowledge deficits about FEPOAs. Education campaigns need not wait for harmonised laws. As noted earlier, there is a basic level of legal consistency around FEPOAs that should be conveyed in public education campaigns as well as explained to older people who seek advice about such instruments.

The AHRC survey revealed public confusion about an attorney's power and responsibilities. Concerningly, many respondents lacked an accurate understanding of the boundaries of the role: they incorrectly considered that an attorney could determine for themselves the duties of their role, rather than being bound by the FEPOA instrument; that an attorney can make decisions extending beyond financial management, such as restricting who can visit an older person in an aged care facility; or that an attorney can assign their role to another person [12]. Understanding these basic rules, and the nature and gravity of the fiduciary duties of the role, are a necessary precondition to better compliance with legal obligations.

An October 2025 legal decision from Western Australia highlights how even a well‐meaning attorney can act in ways that violate their lawful duties [22]. In this case, a niece—who was FEPOA for her 79‐year‐old aunt with significant dementia‐related cognitive impairment—accessed over A$300,000 of her aunt's money to construct a granny flat on the niece's property. Although the ostensible aim was to provide accommodation for the aunt, there was no prior agreement from the aunt to authorise a financial transfer or gift of this nature, and it was clear she lacked capacity to consent to the arrangements. The niece stood to be the main beneficiary, and despite being a supportive carer for her aunt, she failed to understand and avoid the conflict of interest in this situation.

### 5.2 Lack of Professional Advice

According to the AHRC report, only around half of people who made an FEPOA sought professional advice [12, p. 39]. Given the powerful nature of this legal instrument, people should be urged to obtain qualified and personalised advice, especially to ensure careful drafting of a FEPOA tailored to their circumstances. The AHRC survey found that only 27% of people reported considering whether to set limits or conditions on their attorneys [12, p. 39]. This means that a majority of people making FEPOAs are missing the opportunity to exert greater control over how their finances and property are managed in the future.

For example, the FEPOA document can clarify the principal's instructions for giving gifts or donations to specified parties (which would have assisted in the WA case noted earlier), the permissibility of the attorney using funds for their own expenses, and implement third‐party oversight through means, such as an annual audit. In addition to obtaining qualified professional advice, older people should be encouraged to have advance discussions with any proposed attorney. The AHRC survey found few principals had such discussions, echoing prior research findings that older people often appoint family members into these powerful legal roles without adequate planning and shared understanding of the principal's wishes [12, p. 41].

### 5.3 Poor Attorney Selection and Preparation

Careful selection of attorneys is an important defence against future financial abuse. The AHRC survey found that nearly 40% of people who had made a FEPOA had appointed someone with characteristics linked to a higher risk of perpetrating abuse [12]. Such risk factors include dependence on the principal and problems with gambling, alcohol and/or drug use. A July 2025 legal decision in New South Wales highlights a situation where a daughter was considered unsuitable to retain an FEPOA appointment due to financial dependence on her 85‐year‐old mother [23]. Evidence also revealed concerns about the daughter's mental health and controlling behaviour.

Following appropriate attorney selection, people who take on the FEPOA role need preparation and guidance to carry out their duties effectively. There are gaps in practical resources designed to build the capabilities of enduring representatives [24]. For financial attorneys, important skills include financial management and record‐keeping, supporting involvement of the principal in decision‐making, and effective communication with service providers on the principal's behalf.

Proposed model legislative provisions seek to address some of these problems by mandating certain procedures. For instance, when an FEPOA is executed, witnesses would be obliged to give the principal plain language information on rights and obligations under the legal document. The Law Council recommends that a standard FEPOA template should direct a witness to check ‘with the principal whether the attorney has the appropriate attributes’ [19, p. 15]. Appointed attorneys would also be required to sign a statement accepting the role and acknowledging their legal responsibilities. Covering such points at the time of witnessing may, however, be too late in the process, given that the document will already be drafted, and the principal may not wish to revisit their decisions. Moreover, the desired attributes of an attorney and the responsibilities that come with the role need to be more than a ‘tick box’ exercise to achieve the aim of preventing elder financial abuse.

## 6 A Call for Innovation in Research

Calls for harmonisation focus on the perceived drawbacks of differences in legal rules and processes across jurisdictions, but this legislative diversity offers under‐acknowledged opportunities for research. Legislative differences are natural experiments that deserve more regular and rigorous investigation to determine which legal rules warrant inclusion in a model law [25, 26]. We are not proposing that jurisdictions deliberately implement potentially harmful laws to test their effects. Rather, given that variation already exists, there is an opportunity for governments and researchers to collaborate in examining which approaches best protect older people. There is currently a dearth of evidence on the effectiveness of legislative interventions in preventing elder abuse [27, 28]. As a consequence, proposals for model legal provisions are largely based on what intuitively appears to be sensible, rather than on evidence of what works. The developing field of legal epidemiology, ‘the scientific study and deployment of law as a factor in the cause, distribution and prevention of disease and injury in a population’ [29, p. 6], is an important source of methodological guidance for rigorous research [30].

The FEPOA laws in some jurisdictions are currently regarded as offering more stringent safeguards. In New South Wales, a person who witnesses the signing of an FEPOA must have prescribed qualifications or training, such as being a legal practitioner. They must certify that they explained the effect of the FEPOA and the principal appeared to understand the explanation. It is an open question as to whether these enhanced witnessing requirements help to prevent later abuse of the FEPOA role. Contrariwise, it may be that more complex processes for formalising FEPOA documents may deter some older people from making them. Instead, they may rely on informal arrangements that may expose them to a greater risk of exploitation.

Several jurisdictions have legislated specific criminal offences in relation to dishonest conduct by a person seeking or acting in the FEPOA role. Statements on FEPOA forms in some jurisdictions warn appointed attorneys about the risk of civil and/or criminal penalties for non‐compliance with their legal obligations. Harmonisation proposals call for greater consistency in offence provisions and warnings on appointment forms. Although the threat of liability for wrongdoing is a putative safeguard against elder financial abuse, there is little evidence that substantiates the deterrent effect of law [31].

Moreover, insights from the developing field of behavioural jurisprudence emphasise that compliance (or non‐compliance) with the law is powerfully influenced by social norms [31]. For example, in a society with normalised expectations that children deserve to inherit their parents' wealth, a son or daughter appointed as FEPOA might feel entitled to use an ageing parent's assets for their own purposes—the phenomenon known as ‘inheritance impatience’. This social norm of entitlement promotes legal violations. Media stories and advocacy campaigns that seek to raise awareness of elder financial abuse, including by people holding FEPOA, may have unintended consequences. This public messaging may be ‘accidentally sending messages that reinforce the view that the misconduct is normal or commonly accepted’ [31].

We are not alone in urging more research on the legal and behavioural aspects of FEPOAs. The AHRC *Empowering Futures* report identified several important areas for further research, including the role of legal professionals in educating clients and drafting FEPOA documents and the needs and experiences of people living with cognitive impairment and people from diverse cultural and language backgrounds [12]. Little is known about how appointed attorneys carry out their roles, including how they engage in both supported and substitute decision‐making in the context of FEPOAs, and the outcomes of these arrangements.

## 7 Conclusions

Although harmonisation remains an aspirational goal, the substantial difficulties and effort required to achieve greater legislative consistency must be acknowledged. The process will necessitate concerted and ongoing cooperation among states and territories, and harmonisation of FEPOA laws is at the mercy of competing governmental priorities and demands on law‐making resources. The proposed *National Plan to End the Abuse and Mistreatment of Older People* emphasises the need for a ‘national evidence‐based prevention framework’ to inform interventions to end elder abuse [13]. The law is a potent tool to help achieve this goal; however, ambitions for legislative harmonisation and improved experiences and outcomes of FEPOA arrangements are seriously hindered by the lack of evidence.

In regard to the form harmonisation could take, we suggest a model legislation approach—as outlined in Table 1, option b—is most appropriate for FEPOA laws. This approach would provide substantial consistency while allowing jurisdictions to retain or develop provisions that offer enhanced safeguards, informed by evidence and based on local needs. Complete uniformity through referral of powers to the Commonwealth (option c) is unlikely to be achievable given the intersection of FEPOA laws with property, guardianship and other state‐based legal frameworks. However, any harmonisation effort should explicitly incorporate mechanisms for ongoing evaluation, evidence‐sharing between jurisdictions and regular review of model provisions as research evidence accumulates.

Finally, we agree with the ALRC's goal of ‘building trust and confidence in enduring documents as important advance planning tools’ [1]. However, legislative harmonisation alone will not prevent the abuse of FEPOA that harms older people and undermines public trust. Advocacy for harmonisation should not overshadow or stall initiatives to improve the experiences and outcomes for older people who act on their legal rights to make FEPOA appointments. Addressing current deficiencies and poor practices must be a priority.

## Funding

The authors have nothing to report.

## Ethics Statement

The authors have nothing to report.

## Conflicts of Interest

The authors declare no conflicts of interest.

## Data Availability

The authors have nothing to report.
